# A Cell-Adhesive Plasma Polymerized Allylamine Coating Reduces the In Vivo Inflammatory Response Induced by Ti6Al4V Modified with Plasma Immersion Ion Implantation of Copper

**DOI:** 10.3390/jfb8030030

**Published:** 2017-07-20

**Authors:** Uwe Walschus, Andreas Hoene, Maciej Patrzyk, Silke Lucke, Birgit Finke, Martin Polak, Gerold Lukowski, Rainer Bader, Carmen Zietz, Andreas Podbielski, J. Barbara Nebe, Michael Schlosser

**Affiliations:** 1 Research Group of Predictive Diagnostics, Department of Medical Biochemistry and Molecular Biology, University Medical Center Greifswald, 17475 Greifswald, Germany; uwe.walschus@uni-greifswald.de (U.W.); luckes@uni-greifswald.de (S.L.); 2 Department of Surgery, University Medical Center Greifswald, 17475 Greifswald, Germany; hoene@uni-greifswald.de (A.H.); patrzyk@uni-greifswald.de (M.Pa.); 3 Leibniz Institute for Plasma Science and Technology (INP), 17489 Greifswald, Germany; finke@inp-greifswald.de (B.F.); polak@inp-greifswald.de (M.Po.); 4 Institute of Marine Biotechnology, 17489 Greifswald, Germany; lukowski@uni-greifswald.de; 5 Department of Orthopaedics, University Medical Center Rostock, 18057 Rostock, Germany; rainer.bader@med.uni-rostock.de (R.B); carmen.zietz@med.uni-rostock.de (C.Z.); 6 Department of Medical Microbiology, Virology and Hospital Hygiene, University Medical Center Rostock, 18057 Rostock, Germany; andreas.podbielski@med.uni-rostock.de; 7 Department of Cell Biology, University Medical Center Rostock, 18057 Rostock, Germany; barbara.nebe@med.uni-rostock.de

**Keywords:** titanium, copper, antibacterial properties, plasma polymerization, plasma immersion ion implantation, biocompatibility, morphometry, inflammatory response

## Abstract

Copper (Cu) could be suitable to create anti-infective implants based on Titanium (Ti), for example by incorporating Cu into the implant surface using plasma immersion ion implantation (Cu-PIII). The cytotoxicity of Cu might be circumvented by an additional cell-adhesive plasma polymerized allylamine film (PPAAm). Thus, this study aimed to examine in vivo local inflammatory reactions for Ti6Al4V implants treated with Cu-PIII (Ti-Cu), alone or with an additional PPAAm film (Ti-Cu-PPAAm), compared to untreated implants (Ti). Successful Cu-PIII and PPAAm treatment was confirmed with X-ray Photoelectron Spectroscopy. Storage of Ti-Cu and Ti-Cu-PPAAm samples in double-distilled water for five days revealed a reduction of Cu release by PPAAm. Subsequently, Ti, Ti-Cu and Ti-Cu-PPAAm samples were simultaneously implanted into the neck musculature of 24 rats. After 7, 14 and 56 days, peri-implant tissue was retrieved from 8 rats/day for morphometric immunohistochemistry of different inflammatory cells. On day 56, Ti-Cu induced significantly stronger reactions compared to Ti (tissue macrophages, antigen-presenting cells, T lymphocytes) and to Ti-Cu-PPAAm (tissue macrophages, T lymphocytes, mast cells). The response for Ti-Cu-PPAAm was comparable with Ti. In conclusion, PPAAm reduced the inflammatory reactions caused by Cu-PIII. Combining both plasma processes could be useful to create antibacterial and tissue compatible Ti-based implants.

## 1. Introduction

Successful medical use of biomaterials requires a sufficient level of biocompatibility as well as specific physical and chemical properties suitable for the intended application. As established by experience of clinical use for bone replacement, titanium and its alloy Ti6Al4V (Ti) demonstrate good biocompatibility and excellent mechanical strength. While Ti based implants were found to fulfil their intended function for up to ten years after implantation and beyond [1], there is still potential for improvement. Among the most serious implant-related problems, bacterial infections due to the gram-positive species *Staphylococcus aureus* and *Staphylococcus epidermidis* are the primary cause of implant failure [2,3]. Additionally, it has been shown that the protein layer which is initially formed on Ti implants after implantation as part of the implantation-related host reactions renders the surface susceptible to bacterial colonization and the formation of bacterial biofilms [4].

Therefore, the modification of the surface of implants by different coatings to improve their resistance against infections, for example antibiotics, organic or inorganic antimicrobial agents, adhesion-resistant coatings, antibacterial bioactive polymers or nitrogen-monoxide delivering coatings, has been investigated [4]. As an alternative approach, we evaluated a low-temperature plasma-based surface treatment called plasma immersion ion implantation of copper (Cu-PIII), resulting in Cu-releasing Ti surfaces with antibacterial properties as demonstrated by reduction of planktonic and biofilm-attached bacteria [5,6]. The underlying mechanism is the release of Cu ions which were implanted into the titanium oxide layer on top of the Ti surface. However, Cu is also toxic to mammal cells in a concentration-dependent manner, possibly causing adverse tissue effects in vivo. This was, for example, demonstrated in a study using Ti samples with a layer of galvanically deposited Cu which induced stronger acute inflammatory reactions than untreated control samples during the first three days following implantation in rats [7]. Therefore, such adverse effects should be minimized in order to reduce impacts in the peri-implant tissue while still maintaining the antibacterial properties.

This could be achieved by an additional layer with bioactive properties for modulation of tissue-surface interactions. In previous studies, we examined several surface treatments based on plasma polymerized allylamine (PPAAm), resulting in an amino-group rich, positively charged Ti surface characterized by robust anchoring of the PPAAm film with the Ti substrate due to the formation of carbide and oxycarbide bonds, as recently demonstrated by other authors [8]. We were able to show that these PPAAm surfaces had beneficial effects regarding rapid formation of osteoblastic focal adhesions of MG63 cells mediated by paxillin, vinculin and the phosphorylated focal adhesion kinase [9], and were also advantageous for cell morphology and spreading in vitro. Moreover, we were able to demonstrate in a recent in vivo study that, depending on the plasma process parameters, a reduced chronic local inflammatory response was obtained following implantation of PPAAm coated Ti plates in rats [10]. Furthermore, a study on Ti samples treated with a magnetron-sputtered mixed Ti/Cu layer and an additional coating with plasma polymerized ethylenediamine, resulting in an amino-group rich positively charged surface similar to PPAAm, indicated that such cell-adhesive layers could diminish the inflammatory reactions induced by Cu [11]. Interestingly, microbiological experiments in one of our previous studies with *Staphylococcus aureus* cultivation on Cu-PIII-treated Ti samples, either without or with an additional PPAAm film, demonstrated that PPAAm moderately reduced the antibacterial activity of the surface but did not disable it [5]. Thus, an additional coating with PPAAm might be suitable to create a bioactive layer with beneficial effects on the surface of Cu-releasing Ti implants.

Of central relevance for the in vivo biocompatibility of an implant is the inflammatory response, influencing its short- and long-term stability and biofunctionality. Most important among the cells responsible for these reactions are macrophages and other phagocytic cells [12]. Furthermore, T lymphocytes and other immune cells are also involved in implantation-related host reactions [13], although their exact role has not been clarified so far [14,15]. Additionally, mast cells were found to mediate the acute inflammatory response after implantation [16], and recent work demonstrated the infiltration of natural killer (NK) cells in the context of particle-mediated periprosthetic inflammation [17].

Therefore, the aim of this study was to examine the short- and long-term inflammatory in vivo reactions after simultaneous implantation of Ti plates with either a Cu-PIII treatment alone (Ti-Cu) or a combination of a Cu-PIII treatment and an additional PPAAm layer (Ti-Cu-PPAAm) in comparison to untreated Ti control samples in rats. For this, the evaluation of the local inflammatory response by total monocytes/macrophages, tissue macrophages, T lymphocytes, MHC-II^+^ antigen-presenting cells, mast cells and activated NK cells in the peri-implant tissue were morphometrically determined by immunohistochemistry and digital image analysis.

## 2. Results

### 2.1. Physico-Chemical Surface Analysis

The results from ex-situ X-ray photoelectron spectroscopy (XPS) elemental surface analysis, shown in Table 1, demonstrate a comparable elemental composition for Ti-Cu samples and Ti controls (Ti-6Al-4V) regarding the titanium content (Ti-Cu: 12.7% vs. Ti: 16.3%), the carbon content (Ti-Cu: 30.2% vs. Ti: 32.4%), and the oxygen content (Ti-Cu: 45.7% vs. Ti: 44.4%). In contrast, copper was only detected for the Ti-Cu samples with a relative amount of 4.1%. On the other hand, the Ti-Cu-PPAAm samples had a markedly higher content of carbon (74.5%) and nitrogen (23.2%) than the Ti-Cu samples and the Ti controls. Neither titanium nor copper and only 2.2% oxygen were found on the surface of Ti-Cu-PPAAm samples.

**Figure 1:**
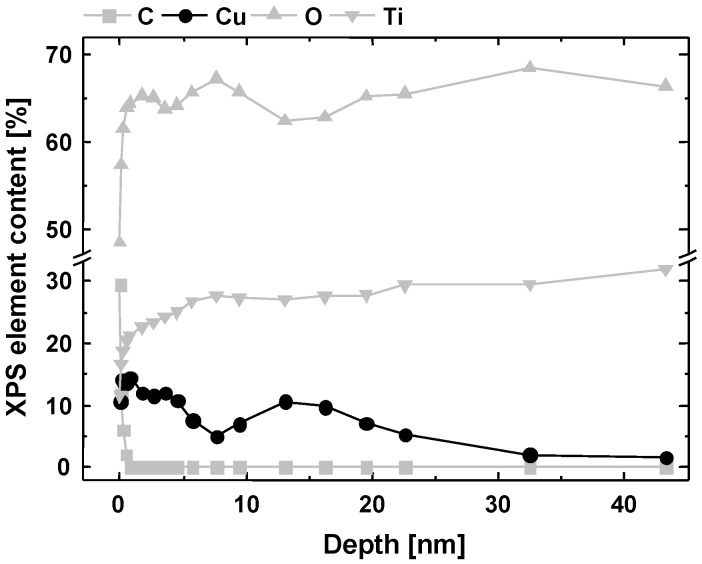
X-ray photoelectron depth profile analysis of a polished Ti6Al4V plate surface treated with plasma immersion ion implantation of copper (Ti-Cu).

Furthermore, the XPS depth profile was analyzed for Ti-Cu samples. Changes of the elemental composition induced by Cu-PIII could be characterized up to a depth of about 45 nm (Figure 1). Cu was implanted underneath the surface, with two maxima for the Cu content at depths of 1.5 nm, i.e., directly on surface level, and around 15 nm, respectively.

### 2.2. Analysis of Copper Release

**Figure 2:**
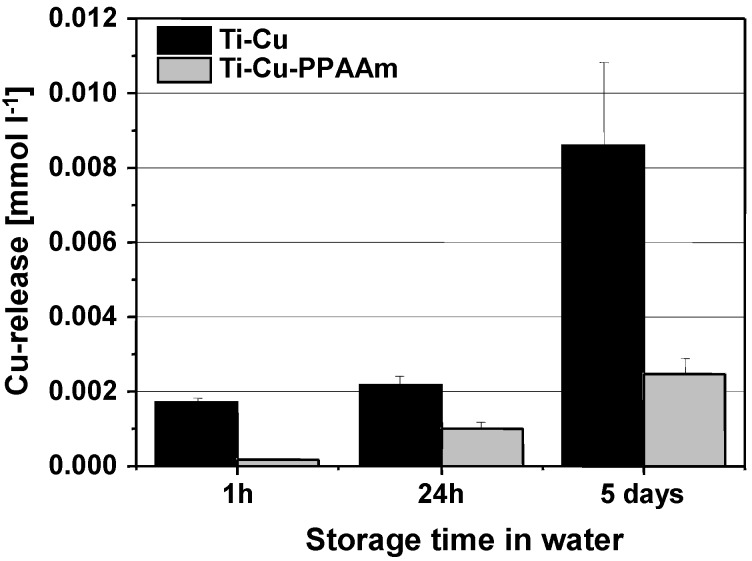
Cu concentrations released from Ti6Al4V plates treated with plasma immersion ion implantation of copper (Ti-Cu, dark bars) or Ti6Al4V plates treated with plasma immersion ion implantation of copper and an additional plasma polymerized allylamine film (Ti-Cu-PPAAm, gray bars) in 2 mL double distilled water over time. Bars represent the mean and whiskers the standard deviation of *n* = 3 different samples for each series.

Cumulative Cu concentrations released from Ti-Cu and Ti-Cu-PPAAm samples in 2 mL double-distilled water over time are summarized in Figure 2. After 24 h, the concentration was 0.0022 ± 0.0002 mmol L^−1^ for Ti-Cu, about twice as much as for Ti-Cu-PPAAm (0.001 ± 0.0002 mmol L^−1^). The difference between both sample series after 5 days was 0.0086 ± 0.0022 mmol L^−1^ for Ti-Cu vs. 0.0025 ± 0.0004 mmol L^−1^ for Ti-Cu-PPAAm.

### 2.3. Morphological Examination

#### 2.3.1. CD68^+^ Total Monocytes/Macrophages (ED1)

**Figure 3:**
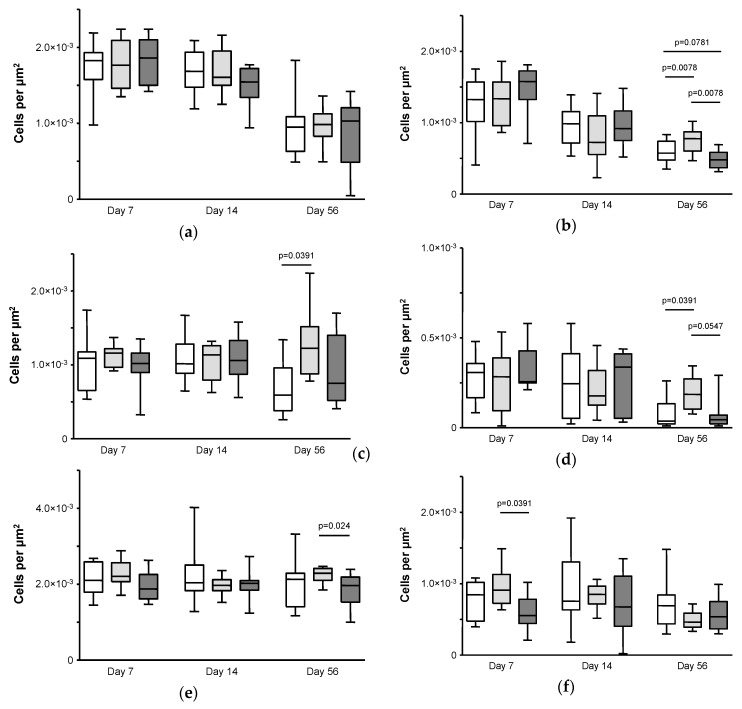
Number of (**a**) CD68^+^ total monocytes and pro-inflammatory macrophages; (**b**) CD163^+^ anti-inflammatory tissue macrophages; (**c**) MHC-II^+^ antigen-presenting cells; (**d**) total T lymphocytes; (**e**) mast cells; and (**f**) activated natural killer cells in the peri-implant tissue of Lewis rats after i.m. implantation of unmodified Ti6Al4V plates (Ti, white boxes), Ti6Al4V plates treated with plasma immersion ion implantation of copper (Ti-Cu, light gray boxes) or Ti6Al4V plates treated with plasma immersion ion implantation of copper and an additional plasma polymerized allylamine film (Ti-Cu-PPAAm, dark gray boxes) after 7, 14 and 56 days. Boxes represent median and interquartile range and whiskers minimum and maximum values; *p*-values indicate differences in pairwise comparison by non-parametric Wilcoxon signed rank test.

For the number of CD68^+^ total monocytes/macrophages (Figure 3a), a significant decrease was observed for all three implant series, for the Ti controls from a median of 1.83 × 10^−3^ cells µm^−2^ (IQR 1.58 × 10^−3^–1.93 × 10^−3^) on day 7 to 0.95 × 10^−3^ cells µm^−2^ (IQR 0.63 × 10^−3^–1.09 × 10^−3^) on day 56 (*p* = 0.0060), for Ti-Cu from a median of 1.77 × 10^−3^ (IQR 1.46 × 10^−3^–2.09 × 10^−3^) on day 7 to 0.98 × 10^−3^ (IQR 0.83 × 10^−3^–1.13 × 10^−3^) on day 56 (*p* = 0.0007), and for Ti-Cu-PPAAm from a median of 1.86 × 10^−3^ (IQR 1.50 × 10^−3^–2.10 × 10^−3^) on day 7 to 1.03 × 10^−3^ (IQR 0.49 × 10^−3^–1.21 × 10^−3^) on day 56 (*p* = 0.0009). There was no significant difference between the implant series on any experimental day.

#### 2.3.2. CD163^+^ Tissue Macrophages (ED2)

Similar to the CD68^+^ total monocytes/macrophages, the number of CD163^+^ tissue macrophages (Figure 3b) decreased significantly for all three implant series, for the Ti controls from a median of 1.33 × 10^−3^ (IQR 1.02 × 10^−3^–1.57 × 10^−3^) on day 7 to 0.57 × 10^−3^ (IQR 0.48 × 10^−3^–0.74 × 10^−3^) on day 56 (*p* = 0.0072), for Ti-Cu from a median of 1.34 × 10^−3^ (IQR 0.96 × 10^−3^–1.57 × 10^−3^) on day 7 to 0.78 × 10^−3^ (IQR 0.60 × 10^−3^–0.87 × 10^−3^) on day 56 (*p* = 0.01), and for Ti-Cu-PPAAm from a median of 1.58 × 10^−3^ (IQR 1.33 × 10^−3^–1.73 × 10^−3^) on day 7 to 0.48 × 10^−3^ (IQR 0.39 × 10^−3^–0.58 × 10^−3^) on day 56 (*p* = 0.0003). While no differences were found between the implant series on experimental days 7 and 14, the Ti-Cu implants had a significantly higher number of CD163^+^ tissue macrophages compared to both the Ti-Cu-PPAAm implants and the Ti controls on day 56 (*p* = 0.0078 respectively). Furthermore, the number of CD163^+^ tissue macrophages for the Ti-Cu-PPAAm implants was lower than for the Ti controls on day 56 (*p* = 0.0781).

#### 2.3.3. MHC-II^+^ Antigen-Presenting Cells (OX6)

For the number of MHC-II^+^ antigen-presenting cells (Figure 3c), no change over time was observed for any of the three implant series. On experimental days 7 and 14, no differences between the implant series were found. However, on day 56 the Ti-Cu implants had significantly higher numbers than the Ti controls (median 1.23 × 10^−3^, IQR 0.88 × 10^−3^–1.52 × 10^−3^ vs. median 0.59 × 10^−3^, IQR 0.38 × 10^−3^–0.96 × 10^−3^; *p* = 0.0391).

#### 2.3.4. T Lymphocytes (R73)

The number of T lymphocytes (Figure 3d) decreased significantly from day 7 to day 56 for the Ti controls, from a median of 0.31 × 10^−3^ (IQR 0.17 × 10^−3^–0.36 × 10^−3^) on day 7 to 0.04 × 10^−3^ (IQR 0.02 × 10^−3^–0.13 × 10^−3^) on day 56 (*p* = 0.0146) and for the Ti-Cu-PPAAm implants, from a median of 0.27 × 10^−3^ (IQR 0.25 × 10^−3^–0.43 × 10^−3^) on day 7 to 0.05 × 10^−3^ (IQR 0.02 × 10^−3^–0.07 × 10^−3^) on day 56 (*p* = 0.0175), but not for the Ti-Cu implants. Consequently, the Ti-Cu implants had significantly higher numbers of T lymphocytes on day 56 (median 0.18 × 10^−3^, IQR 0.10 × 10^−3^–0.27 × 10^−3^) in comparison to the Ti controls (*p* = 0.0391) and to the Ti-Cu-PPAAm implants (*p* = 0.05), while there was no significant difference between the three implant series on days 7 or 14.

#### 2.3.5. Mast Cells (AD1)

The numbers of mast cells (Figure 3e) remained unchanged over the course of the study for all three implant series. On day 56, the Ti-Cu implants had significantly higher numbers of mast cells in comparison to the Ti-Cu-PPAAm implants (median 2.29 × 10^−3^, IQR 2.10 × 10^−3^–2.42 × 10^−3^ vs. median 1.97 × 10^−3^, IQR 1.53 × 10^−3^–2.19 × 10^−3^; *p* = 0.024).

#### 2.3.6. Activated NK Cells (ANK61)

For the numbers of activated NK cells (Figure 3f), a significant decrease was observed only for the Ti-Cu implants, from a median of 0.91 × 10^−3^ (IQR 0.72 × 10^−3^–1.13 × 10^−3^) on day 7 to 0.46 × 10^−3^ (IQR 0.39 × 10^−3^–0.59 × 10^−3^) on day 56 (*p* = 0.0016), but not for the other two implant series. This was mainly due to an elevated number of activated NK cells for the Ti-Cu implants in the early phase, significantly higher than for the Ti-Cu-PPAAm implants with a median of 0.55 × 10^−3^ (IQR 0.44 × 10^−3^–0.78 × 10^−3^) on day 7 (*p* = 0.024), but also with a lesser extent compared to the Ti controls. On days 14 and 56, no differences were found between the three implant series.

## 3. Discussion

Anti-infective biomaterials could improve the clinical outcome following implantation since bacterial infections are a leading cause for implant failure. Previous in vitro studies showed antibacterial properties for Ti6Al4V (Ti) surfaces either treated with plasma immersion ion implantation of copper (Cu-PIII) alone or with Cu-PIII followed by an additional plasma polymerized allylamine (PPAAm) film [5,6]. Earlier experiments also demonstrated that PPAAm is advantageous regarding rapid focal adhesion formation, morphology and the spreading of MG63 cells in vitro [9], with no negative influence on the local tissue reactions and a reduced chronic inflammatory response in vivo [10]. Consequently, the present study aimed at evaluating the in vivo effects of an additional PPAAm film on Cu-PIII-treated Ti plates regarding the acute and chronic local inflammatory reactions.

XPS demonstrated that Ti-Cu samples were overall comparable to Ti controls for relative amounts of titanium, carbon, oxygen and nitrogen. XPS also confirmed Cu incorporation by Cu-PIII into the TiO_2_ layer and Ti itself. In comparison, Ti-Cu-PPAAm samples had higher amounts of carbon and nitrogen as PPAAm films consist of a cross-linked plasma-polymer nanolayer with a hydrocarbon network containing nitrogen functional groups such as amines, acid amides, imines and nitriles [18]. Furthermore, neither Ti nor Cu and only a small oxygen amount were detected on Ti-Cu-PPAAm samples, indicating complete coverage by PPAAm with a thickness of about 50 nm. The XPS depth profile revealed two peaks for the Cu distribution, one at a depth of about 1.5 nm and a second around 15 nm. The first peak is probably due to coating processes with low energetic Cu ions, while the second peak is the actual implantation peak. However, it should be noted that the measured depth is slightly too high for implantation of 10 keV Cu ions into Ti as computer simulations with the software system TRIM 2013 (‘Transport of Ions in Matter’; (C) 1984–2013, James F. Ziegler) gave a simulated mean depth of about 10 nm. The difference can be mainly explained by two effects, on the one hand the Cu coating on top of the surface and on the other hand by the measuring method itself (XPS with depth profiling). The Ar ions used for depth profiling have an energy of 4.5 keV. They not only induce surface sputtering but also hit the implanted Cu atoms inside the titanium, thereby driving them deeper into the substrate. Furthermore, the photoelectrons from the surface measured with XPS are an integration over the depth of up to 5 nm (mean free path of electrons in titanium).

Subsequent examination of the Cu release from Ti-Cu and Ti-Cu-PAAAm samples however indicated that PPAAm inhibits the Cu^2+^ diffusion process to a certain degree. Moreover, released Cu^2+^ concentrations from the polished Ti surfaces used in the present study were also lower than, for example, for rougher corundum blasted surfaces treated by Cu-PIII or dual high power impulse magnetron sputtering (dual HiPIMS) [19]. Furthermore, the Cu release results are consistent with earlier microbiological experiments in which both Ti-Cu and Ti-Cu-PPAAm samples reduced residual *Staphylococcus aureus* numbers by at least one order of magnitude after 24 h in contrast to Ti controls, with a moderate reduction of antibacterial effectiveness due to the additional PPAAm film [5]. By comparison, samples coated with Cu by dual HiPIMS had a stronger antimicrobial effect than Ti-Cu samples as used in the present study, which also showed a reduced cytotoxicity to MG-63 osteoblasts compared to the dual HiPIMS samples [19]. This underlines the importance of optimizing the Cu release kinetics regarding the balance between antibacterial and cytotoxic effects by choosing a suitable plasma treatment with appropriate process conditions.

Following physico-chemical evaluation, simultaneous intramuscular implantation of Ti-Cu, Ti-Cu-PPAAm and Ti control samples into the neck musculature of rats was performed for comparative in vivo examination of their effects on acute and chronic local inflammatory reactions. This experimental model, resembling the clinical situation of a predominantly muscular environment for orthopaedic Ti implants, was established and optimized in several recent studies [7,10,11,20,21]. It can be performed with minimal surgical complications and post-operative stress, which are relevant considerations for animal welfare. Furthermore, musculature is well supplied with blood and thus particularly suitable to study inflammatory tissue reactions. Simultaneous implantation of different samples into the same experimental animals also facilitates an intra-individual comparison, reducing both the number of animals required as well as the broad individual variability regarding inflammatory and immunological reactions as observed in earlier studies [22,23].

Overall, the histological results indicate a shift from acute to chronic inflammation for all three sample series. This is especially demonstrated by the decrease over time for numbers of CD68^+^ total monocytes/pro-inflammatory macrophages (ED1) and anti-inflammatory CD163^+^ tissue macrophages (ED2) as macrophages play a major role in biomaterial-related inflammation [12]. In the early acute phase of inflammation at day 7 the Ti-Cu implants induced the strongest reaction only for the activated NK cells, which possess no antigen-specific receptors and are part of the innate immune response. In the late chronic phase of inflammation at day 56, tissue reactions were strongest for Ti-Cu samples for the CD163^+^ tissue macrophages, the MHC-II^+^ antigen-presenting cells, the T lymphocytes and the mast cells. These late phase observations, not seen for CD68^+^ total monocytes/pro-inflammatory macrophages and activated NK cells, possibly indicate a stronger involvement of immunological reactions induced by Ti-Cu implants in the chronic inflammation. Furthermore these results clearly demonstrate beneficial effects from an additional PPAAm film by reducing the long-term chronic inflammatory tissue response induced by Cu-PIII. This is consistent with earlier results regarding another amino-group rich coating, plasma polymerized ethylendiamine (PPEDA), on the inflammatory reactions caused by Ti samples with a magnetron-sputtered Cu layer [11]. It should be noted that the Cu-PIII treatment results in implantation of Cu into both the TiO_2_ layer and Ti itself while a mixed layer of atomic Cu and Ti is deposited onto the sample surface by dual HiPIMS as used in that recently published study [19]. Furthermore, the precursor ethylenediamine used for PPEDA coating has two amino groups versus one amino group for allylamine used for PPAAm coating, resulting in a different amino-group density. Another study using Ti samples treated with plasmaelectrolytical oxidation followed by galvanic Cu deposition revealed increased inflammatory reactions for Cu-treated samples compared to controls already during the acute phase [7]. Thus, despite the different Cu deposition methods used in these studies and corresponding physico-chemical differences between the resulting samples, as well as between the cell-adhesive films PPAAm and PPEDA, the overall effects on the inflammatory tissue response were notably comparable.

In summary, an additional PPAAm film reduced the chronic local inflammatory reactions caused by Cu-PIII-treated Ti implants. This observation extends earlier in vitro results concerning their antibacterial activity [5,6] as well as in vitro and in vivo studies on PPAAm as single cell-adhesive coating for Ti implants [9,10] and electronspun poly(l-lactide-co-d/l-lactide) meshes [24]. The combination of plasma immersion ion implantation of copper and plasma polymerized allylamine could thus be used for implants with both antibacterial and tissue compatible properties, aimed at preventing short-term infections while maintaining optimal tissue integration. However, the results also demonstrate that further studies are needed to find the optimal adjustment between these two aspects. Such studies could include the examination of additional aspects of the tissue response and cellular behavior aimed at providing a deeper understanding of the inflammatory response beyond the comparison between different surface modifications in the present study. Possible parameters include for example the expression of immune phenotype markers, the levels of reactive oxygen species (ROS), a differentiation of the T cell response into subpopulations such as helper T cells, cytotoxic T cells, or regulatory suppressor T cells, a closer look at pro- and anti-inflammatory markers like expression of different cytokines, or a Masson’s trichrome staining to examine the extent of fibrosis. Such investigations could provide information on how different aspects of the short- and long-term inflammatory response on the one hand affect the implant performance and on the other hand are influenced by certain material properties, revealing important information regarding cell-material interactions and structure-property-function relationships which might also be relevant for other materials or surface modifications.

More comprehensive studies are also needed to fully assess the extent and duration of the antibacterial effects of samples treated with Cu-PIII and PPAAm. The respective data in our previous study [5] covers only a short period of incubation with a small inoculation volume, which did not fully cover the samples. This experimental approach primarily had the short phase immediately after implantation in mind, which is often described as a ‘race for the surface’ between host cells and bacteria [25]. During this short period, impeding initial bacterial adhesion and colonization is especially important to prevent formation of a biofilm which is nearly impossible to remove in clinical practice. Additional experiments including extended incubation periods under liquid and flow-based conditions to better mimic the in vivo microenvironment are necessary to examine whether the antibacterial effectiveness corresponds to the in vitro release data and to address the long-term effects of the Cu-PIII/ PPAAm treatment on adherence, survival and biofilm formation of bacteria as well as their possible adjustment and selection. Furthermore, in vivo experiments using implants with spiked bacterial loads might help to evaluate the in vivo efficacy as well as the influence of bacterial infections on the tissue response, like the extent of fibrosis.

## 4. Materials and Methods 

### 4.1. Samples and Plasma Treatment

#### 4.1.1. Samples

Plasma treatments were carried out on either polished Ti6Al4V discs (diameter 11 mm, height 1 mm) for physicochemical surface analyses or small square Ti6Al4V platelets (5 × 5 × 1 mm^3^) for in vivo investigations, with a defined arithmetic roughness of R_a_ = 0.19 µm or R_a_ = 0.28 µm respectively (all samples obtained from DOT GmbH, Rostock, Germany).

#### 4.1.2. Plasma Immersion Ion Implantation of Copper (Cu-PIII)

Plasma Immersion Ion Implantation of copper (Cu-PIII) was performed in an ultra-high vacuum (UHV) reactor (constructed in-house at the Leibniz Institute for Plasma Science and Technology, Greifswald, Germany) with a capacitively coupled radio frequency-discharge (13.56 MHz) and two coplanar copper electrodes. The pulsed voltage applied was at 10 kV and the working pressure at 2 Pa. The pulses for the Cu-PIII had a short rise time in the range of 200 ns, a repetition rate of 1 kHz and pulse length of 2 µs. These parameters induced a mean implantation current of about 7 mA. The temperature of the sample attains about <50 °C. The Cu-PIII treatment was carried out on each sample side for 55 min. The resulting samples are named Ti-Cu.

#### 4.1.3. Creation of Plasma Polymerized Allylamine Films on Ti-Cu Surfaces (Ti-Cu-PPAAm)

On one series of Ti-Cu samples, an additional plasma polymerized allylamine (PPAAm) thin film was deposited in a microwave (MW, 2.45 GHz) plasma reactor V55G (Plasma-finish GmbH, Schwedt/Oder, Germany) in a two-step procedure without breaking the vacuum. At first, all samples were decontaminated and activated by a continuous wave oxygen/argon plasma (500 W, 50 Pa, 100 sccm O_2_/25 sccm Ar) and secondly followed by the deposition of the PPAAm thin film (~50 nm thick) (500 W, 50 Pa, 50 sccm allylamine/50 sccm Ar; pulsed plasma regime 0.3 s on/1.7 s off). The overall plasma on time was 144 s and the total processing time 960 s. The PPAAm plasma deposition process was performed on each sample side. The resulting samples are named Ti-Cu-PPAAm.

### 4.2. Physico-Chemical Analysis

#### 4.2.1. X-ray Photoelectron Spectroscopy (XPS) Analysis

The elemental chemical surface composition and chemical binding properties of the different implant series were examined by XPS with an AXIS ULTRA spectrometer (Kratos, Manchester, UK) as previously described in detail [5]. In summary, the monochromatic Al Kα line at 1486 eV (150 W) was used with implemented charge neutralization and a pass energy of 80 eV for estimating the chemical elemental composition or of 10 eV for highly resolved peaks. The C-C/C-H component of the C 1s peak was adjusted to 285 eV [26]. Each measurement was repeated three times at different surface positions of the same sample.

XPS depth profiles were recorded by sputtering with Ar^+^ ions with a kinetic energy of 4.5 keV for a specific time. After a certain sputtering time, the ion flux was interrupted and the elemental composition of the actual surface was determined. Afterwards the next sputtering cycle was performed. The software system CasaXPS version 2.3.15 (Casa Software Ltd, Teignmouth, UK) was used for quantification.

#### 4.2.2. Measurement of PPAAm Film Thickness

Measurement of PPAAm film thickness was performed using the surface profiler Dektak 3ST (Veeco, Plainview, NY, USA) as described previously [9]. Briefly, the silica wafer was partially coated before plasma treatment with a cellulose acetate film which was removed after PPAAm film deposition, taking along the plasma polymer situated thereon. The film thickness was subsequently determined with the surface profiler (stylus tip radius: 2.5 mm) as the level difference of the step created by removal of the cellulose acetate film, based on the mean of 25 measurement points on each side of the step.

#### 4.2.3. Copper Release Measurement

The copper release from Ti-Cu and Ti-Cu-PPAAm samples was measured by storing the samples in 2 mL double-distilled water (TKA Wasseraufbereitungssysteme GmbH, Niederelbert, Germany) for five days at room temperature. Cu concentrations in storage liquid were measured by means of atomic absorption spectrometry (AAS) ZEEnit 650 (Analytik Jena AG, Jena, Germany) with electro-thermal atomisation. Three separate samples from each series were analyzed.

### 4.3. In Vivo Experiments

#### 4.3.1. Laboratory Animals

24 male Lewis rats (age 100 days, mean weight 356 ± 11 g) were kept in in-house facilities of the University Medical Center Greifswald under conventional housing and feeding conditions. All animal experiments were performed in accordance with the animal protection law of the Federal Republic of Germany in its new version of 1 January 1987, with the principles of care for animals in laboratories (drawn up by the National Society for Medical Research) and with the Guidelines for Keeping and Using Laboratory Animals (NIH Publication No.80-23, revised 1985). The study was reviewed and approved (approval code 7221.3-1.1-074/11) by the State Office for Agriculture, Food Safety and Fishery of the federal state of Mecklenburg-Vorpommern (Rostock, Germany).

#### 4.3.2. Implantation Procedure and Tissue Sampling

The animals were anesthetized by i.p. application of a mixture of Rompun^®^ (Bayer, Leverkusen, Germany) and Ketamin^®^ (Sanofi-Ceva, Düsseldorf, Germany) with dosing relative to individual body weight. Three implant samples were simultaneously implanted in each animal into small intramuscular pockets in the neck musculature: one implant treated with plasma immersion ion implantation of copper only (Ti-Cu), one implant treated with plasma immersion ion implantation of copper and an additional plasma polymerized allylamine coating (Ti-Cu-PPAAm) and one untreated Ti6Al4V control implant (Ti). The three implants were separated by at least 2 cm from each other in a triangular alignment and fixed in their respective tissue pockets with a nonresorbable synthetic polypropylene suture (PROLENE^®^; Ethicon Endo-Surgery, Inc., Hamburg, Germany).

Eight randomly selected animals were euthanized after 7, 14 and 56 days, and the implants with a sample of the surrounding tissue were carefully explanted after surgical opening of the implantation site. After immediate freezing of the samples with laboratory freezer spray New Envi-Ro-Tech^TM^ (Thermo Electron Corporation, Pittsburgh, PA, USA), they were cut with a scalpel with the section plane at right angle with the implants after which the implants were carefully removed from the frozen tissue using tweezers. The embedding medium Shandon Cryomatrix^TM^ (Thermo Electron Corporation, Pittsburgh, PA, USA) was used to fill the remaining tissue pockets to preserve their form during further processing. The tissue samples were subsequently shock frozen and stored at −80 °C.

### 4.4. Morphological Examination

#### 4.4.1. Immunohistochemistry and Histochemistry

A Cryotome 2800 Frigocut N (Reichert-Jung, Nussloch, Germany) was used to prepare frozen tissue sections (thickness: 5 µm). The following antibodies were used for immunohistochemical staining according to the manufacturer’s protocols: ED1 for CD68^+^ monocytes/macrophages, ED2 for CD163^+^ tissue macrophages, R73 for T lymphocytes, OX6 for MHC-II^+^ antigen-presenting cells (all antibodies obtained from MorphoSys AbD Serotec GmbH, Duesseldorf, Germany), AD1 for mast cells (BD Biosciences, Heidelberg, Germany) and ANK61 for activated natural killer (NK) cells (Santa Cruz Biotechnology, Heidelberg, Germany). Detection of bound primary antibodies was performed with the Alkaline Phosphatase Anti-Alkaline Phosphatase method (APAAP; DakoCytomation GmbH, Hamburg, Germany), the polyclonal rabbit anti-mouse-immunoglobulin (Z259; Dako DenmarkA/S, Glostrup, Denmark) and the chromogenic substance new fuchsine.

#### 4.4.2. Microscopic Equipment

The stained histological samples were evaluated using a light microscope CX41 (Olympus, Hamburg, Germany) at a visual magnification of 100×. A colour camera DP20 (resolution 1600 × 1200 Pixel, CCD size 1/1.8 in; Olympus, Hamburg, Germany) attached via a camera adapter U-TV0.63XC (adapter magnification 0.63×; Olympus, Hamburg, Germany) was used to obtain digital images. Images were taken with an objective magnification of 10× and represent an area of about 1 mm^2^ tissue section at the given camera resolution.

#### 4.4.3. Image Analysis Procedure

The image analysis program ImageJ version1.44 (U.S. National Institutes of Health, Bethesda, MD, USA) [27] and the software plugins Grid and CellCounter were used to determine the number of positively stained cells in defined areas [28]. Briefly, a grid with a square size of 20,000 pixels was superimposed onto the images, and five representative squares directly adjacent to the implant pockets were examined. If necessary, the area covered by artefact zones and other regions without tissue was measured and deducted from the total analyzed area of 100,000 pixels per image. One pixel corresponded to an area of 0.4796 µm^2^ in the chosen microscopic magnification, based on evaluation of a microscopic slide with a printed length scale. Final results given as positively stained cells per µm^2^ represent the average counts from two independent investigators.

### 4.5. Statistical Data Analysis

Data of copper release measurements are given as mean concentration values ± standard deviation of analysis. For all morphological analysis, cell count data are given as median cells per µm^−2^ section plane and interquartile range (IQR) with whiskers as minimum and maximum individual cell numbers. The non-parametric Wilcoxon signed rank test was used for pairwise comparison of the number of positively stained cells in the peri-implant tissue on the same experimental day. Results for the same implant type over the course of the three experimental days were analyzed with the non-parametric Kruskal-Wallis test. A *p*-value of less than 0.05 was considered to be statistically significant for all tests. The software system GraphPad Prism version 4.03 (GraphPad Software, Inc., San Diego, CA, USA) was used for statistical analysis.

## Figures and Tables

**Table 1:** Elemental surface composition of unmodified Ti6Al4V plates (Ti), Ti6Al4V plates treated with plasma immersion ion implantation of copper (Ti-Cu) or Ti6Al4V plates treated with plasma immersion ion implantation of copper and an additional plasma polymerized allylamine film (Ti-Cu-PPAAm) determined by X-ray photoelectron spectroscopy (XPS); data are given as mean and standard deviation of measurements at *n* = 3 different surface positions of the same sample.

Implant Series	Ti [%]	Al [%]	V [%]	Cu [%]	C [%]	O [%]	N [%]
Ti	16.3 ± 0.2	2.5 ± 0.4	0.4 ± 0.1	0	32.4 ± 0.9	44.4 ± 0.9	2.7 ± 0.3
Ti-Cu	12.7 ± 0.2	0.7 ± 0.04	0.4 ± 0.03	4.1 ± 0.1	30.2 ± 1.3	45.7 ± 1.3	0
Ti-Cu-PPAAm	0	0	0	0	74.5 ± 0.3	2.2 ± 0.1	23.2 ± 0.2
